# Manganese: From Soil to Human Health—A Comprehensive Overview of Its Biological and Environmental Significance

**DOI:** 10.3390/nu16203455

**Published:** 2024-10-11

**Authors:** Sarfo Kwaku Obeng, Martin Kulhánek, Jiří Balík, Jindřich Černý, Ondřej Sedlář

**Affiliations:** Department of Agro-Environmental Chemistry and Plant Nutrition, Faculty of Agrobiology, Food and Natural Resources, Czech University of Life Sciences, 165 00 Prague, Czech Republic; obeng@af.czu.cz (S.K.O.); balik@af.czu.cz (J.B.); cernyj@af.czu.cz (J.Č.); sedlar@af.czu.cz (O.S.)

**Keywords:** manganese, Mn cycling in environment, food chain, water quality, Mn metabolism, human nutrition

## Abstract

**Background:** Manganese is an essential micronutrient that plays a pivotal role in environmental systems, plant physiology, and human health. This review comprehensively examines the manganese cycle in the environment, its absorption and transport mechanisms in plants, and the implications of manganese exposure to human health. **Objectives:** The objectives of this review are to (i) analyze the environmental cycling of manganese and its bioavailability, (ii) evaluate the role of manganese in plant metabolism and disease resistance, and (iii) assess the impact of manganese toxicity and deficiency on human health. **Conclusion:** This review highlights that while manganese is crucial for photosynthesis, enzyme activation, and resistance to plant diseases, both its deficiency and toxicity can have severe consequences. In plants, manganese deficiency can lead to impaired growth and reduced crop yields, while toxicity, particularly in acidic soils, can inhibit photosynthesis and stunt development. In humans, manganese is necessary for various physiological processes, but overexposure, especially in occupational settings, can result in neurodegenerative conditions such as manganism. The conclusion emphasizes the importance of managing manganese levels in agriculture and industry to optimize its benefits while minimizing health risks. A multidisciplinary approach is advocated to enhance agricultural productivity and ensure public health safety.

## 1. Introduction

Earth’s 12th most abundant element and fifth most abundant metal is manganese. This silver-grey metal oxidizes easily. Thus, Mn is present in oxides, carbonates, and silicates. Mn occurs in positive oxidation states (+2, +3, +4, +6, and +7) despite its negative oxidation state (−3). Mn^2+^ and Mn^3+^ are mainly oxidized in living organisms. Mn^2+^ is the most stable form, whereas Mn^3+^ is a potent oxidant that generally disproportionates to Mn^2+^ and Mn^4+^ or combines with proteins such as transferrin (Tf) [1]. Every year, natural earth erosion releases tonnes of Mn into the air, soil, and rivers for microbes, plants, and animals to absorb [2].

Manganese is a micronutrient necessary for most living organisms. It is crucial in biological clusters as an enzyme cofactor and catalytic metal. Manganese is one of the most studied micronutrients for plant disease effects and is essential for root and foliar disease resistance [3,4,5,6]. Its availability in soil depends on environmental and biotic factors [7]. Plants require manganese more than fungus and bacteria, therefore the pathogen may benefit [8].

As a component of photosynthesis, manganese is an important element in plant metabolism. It is a structural component of the photosystem II water-splitting protein and also stores and delivers electrons to chlorophyll reaction centers. Furthermore, Mn is an important metallic component of many enzymes, including arginase, glutamine synthetase, pyruvate carboxylase, and manganese superoxide dismutase (MnSOD). Plant and human manganese deficiency has been widely studied. Manganese is needed for several metabolic activities in human nutrition, including optimal growth [9,10].

Mn is an essential mineral in human nutrition, playing a crucial role in several physiological processes. As mentioned below in more detail, it is necessary for the functioning of enzymes involved in metabolism, bone formation, and regulation of blood sugar levels. It also supports the antioxidant defense system by contributing to the activity of superoxide dismutase. While it is important for overall health, the body requires only small amounts, with the recommended intake for adults being around 3.5 to 7.0 mg per day [11].

On the other hand, Mn intoxication, or poisoning, poses a risk when exposure exceeds the ability to regulate it. Chronic overexposure can appear mostly via inhalation and can lead to the disease called manganism, a neurological disorder with symptoms similar to those of Parkinson’s disease [12,13,14].

The purpose of this review is to provide a comprehensive overview of manganese’s biological and environmental importance. It deals with the manganese cycle in the environment, focusing on its mobility, participation in plant metabolism, and influence on human health and behavior. It emphasizes the necessity for balanced Mn management in the environment and thus mitigating health risks.

## 2. The Manganese Cycle

**Figure 1:**
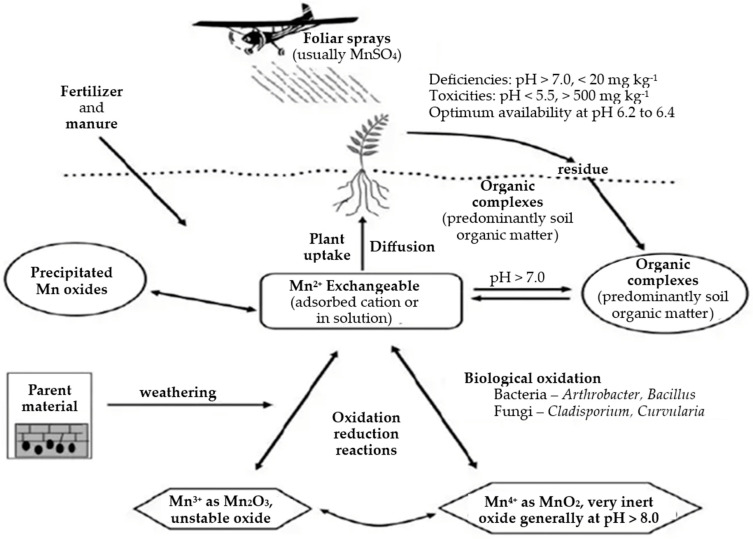
Cycle of manganese in the environment [16].

Figure 1 shows the manganese cycle, which indicates that microorganisms are key mediators in Mn oxidation in a variety of environments. Microbial Mn oxide minerals are typically dark brown to black and poorly crystalline with birnessite (layered) or todorokite (tunnel) crystal structures. Both bacteria and fungi produce Mn oxide minerals, although the exact mechanism for Mn oxidation remains elusive [15].

Chemolithoautotrophic Mn oxidation is highly unlikely to be carried out with the enzymes currently known, although indirect oxidation of Mn during heterotrophic growth or reproduction has been observed in both bacteria and fungi [15]. Differences in Mn availability influence not only the microbial community structure associated with ferromagnetic deposits but also Mn cycling and microbial functions [17,18].

### 2.1. Manganese in Environment

#### 2.1.1. Manganese Levels in Soil

Manganese (Mn) is the 10th most common element in the Earth’s crust, where manganese-containing compounds are second in quantity to iron (Fe). The total quantity of manganese in soil ranges between 20 and 3000 mg kg^−1^, with an average of 600 mg kg^−1^. Divalent manganese (Mn^2+^) is absorbed by clay minerals and organic material, and this form is the most significant in plant nutrition [19]. Manganese occurs in soil as exchangeable manganese, manganese oxide, organic manganese, and as a component of ferro-manganese silicate minerals. The manganese ion (Mn^2+^) is similar in size to magnesium (Mg^2+^) and ferrous iron (Fe^2+^) and can replace these elements in silicate minerals and iron oxides. Soil manganese reactions are highly complicated. Soil pH, organic matter, moisture, and aeration are usually the key factors influencing manganese bioavailability [20].

#### 2.1.2. Manganese in Water

The oxidation of manganese (Mn^2+^) compounds in aqueous solutions leads to the precipitation of manganese, which subsequently causes encrustation issues. According to Bean et al. [21], manganese can create deposits on water pipes, even at concentrations as low as 0.02 mg L^−1^. These deposits have the potential to detach from the pipes and manifest as a black precipitate. Several countries have established a threshold of 0.05 mg L^−1^ as the standard for manganese concentration, beyond which issues related to discoloration may arise [22].

Manganese is naturally present in numerous surface water and groundwater sources, as well as in soils that have the potential to erode and contribute to the aforementioned water bodies. Nevertheless, it is important to acknowledge that anthropogenic activities (mining, steel production, manufacturing) play a significant role in the introduction of manganese contamination into water sources within certain regions. The literature indicates that ambient levels of manganese in seawater have been documented to vary between 0.4 and 10 µg L^−1^ [23], with an average concentration of approximately 2 µg L^−1^ [19]. The concentration of levels in fresh water generally spans from 1 to 200 µg L^−1^, as reported by Barceloux and Barceloux [24]. According to a report by the Agency of Toxic Substances and Disease Registry [23], a survey conducted on river water in the United States revealed the presence of dissolved manganese at a concentration of 51 µg L^−1^. Since 1991, the National Water Quality Assessment Program of the US Geological Survey has collected a restricted amount of data pertaining to representative study basins across the United States. The data presented in the study by Leahy and Thompson [25] and the report by the US Geological Survey [26] demonstrate that surface waters exhibit a median manganese level of 16 µg L^−1^ with 99th-percentile concentrations ranging from 400 to 800 µg L^−1^. Industrial pollution is commonly linked to elevated levels of aerobic waters. The presence of reducing conditions in groundwater and certain bodies of water promotes elevated levels of manganese. Studies have reported concentrations of up to 1300 µg L^−1^ in neutral groundwater and 9600 µg L^−1^ in acidic groundwater [23]. Using data from the National Water Quality Assessment Program, it has been observed that the 99th percentile level of manganese in groundwater is generally higher (5600 µg L^−1^) compared with surface water. However, the median level of manganese in groundwater (5 µg L^−1^) is lower than that in surface water, as reported by the US Geological Survey [26].

## 3. Manganese in Plants

### 3.1. Plant Manganese Transport and Accumulation

As previously stated, the only accessible metal form for plants is reduced Mn (Mn^2+^). It is taken up by an active transport pathway in epidermal root cells and transferred into plants as the divalent cation Mn^2+^ [8,27,28]. The absorption of manganese by roots is a biphasic process. Mn^2+^ and Ca^2+^ and other cations readily interchange in the rhizosphere during the first and fast absorption phase, which is reversible and non-metabolic. Mn^2+^ seems to be absorbed by the negatively charged cell wall elements of root cell apoplastic gaps during this phase [29,30]. The second phase is slower, with Mn^2+^ being more difficult to exchange. Plant metabolism is required for its absorption into the symplast [31], although the particular pathways are unknown [29]. Kinetic tests have shown that Mn transport rates are 100 to 1000 times greater than the predicted plant demand for this element [30,32]. The large capacity of ion carriers and channels in Mn ion transport via the plasma membrane at a rate of several hundred to several million ions per second per protein molecule explains these transport rates [29].

Transpiration (xylem) stream transport moves from the roots to the above-ground sections of plants, while phloem transport is more selective, occurring from sources to sinks [8]. Nonetheless, little phloem mobility has been documented for Mn, and its redistribution may be dependent on plant species and developmental phases [33]. Indeed, it has been found that at the mature stage of wheat, Mn transfer from roots to grains is usually inadequate. Mn mobility in the phloem is rather low, emphasizing the relevance of xylem in the transfer of this element, especially in wheat grain discharge [34].

### 3.2. Manganese’s Biochemical Role in Plants

Because divalent manganese ions (Mn^2+^) are readily transformed to Mn^3+^ or Mn^4+^ and vice versa, manganese plays an essential role in oxidation and reduction processes, as well as electron transport in photosynthesis. Furthermore, manganese works as an activator of several enzymes (approximately 35 distinct enzymes). Manganese is essential for the activation of numerous enzymes involved in oxidation processes, carboxylation, carbohydrate metabolism, phosphorus reactions, and the citric acid cycle. Protein manganese in photosystem II and MnSOD might be mentioned as two of the most significant enzymes. More than 90% of superoxide dismutase is found in chloroplasts, with the remaining 4–5% found in mitochondria [10,35,36,37]. Mn^2+^ also activates dehydrogenase and decarboxylase in the Krebs cycle (TCA) [8,38]. It is needed for the formation of chlorophyll and is required by photosystem II, which is also involved in cell division and plant development. Manganese activates RNA polymerase. It plays an important role in lipid metabolism, and since it is involved in nitrate reduction enzymes, nitrates build up in manganese-deficient leaves. Furthermore, the amount of lignin in the plant will decrease due to manganese deficiency, with this reduction being more severe in the roots; this matter is critical, particularly in reducing resistance to fungi infecting the plant roots [8,35,39,40].

### 3.3. Manganese Deficiency in Plants

**Figure 2:**
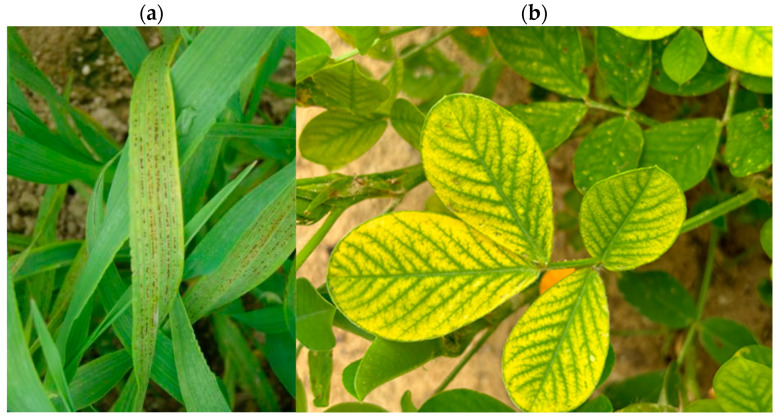
(**a**) Typical interveinal necrosis caused by manganese deficiency in barley [49]; (**b**) Manganese deficiency in peanuts [50].

Manganese deficiency (Figure 2a,b) is broad in terms of geographical distribution, although it is more common in calcareous soils, soils with high pH (arid and semi-arid parts of the globe), and notably soils with inadequate aeration. There is also a manganese deficit caused by soil’s surface erosion [41].

In general, soil organic matter influences the quantity of dissolved manganese in the soil. Manganese levels in certain podzolic soils are naturally low, owing to extensive leaching [41]. Manganese solubility decreases with rising pH; it decreases 100-fold with an increase of one unit of pH. Chloroplasts are the cell components most sensitive to manganese deficiency; in fact, manganese deficiency dramatically damages chloroplast structure. Manganese shortages reduce net photosynthesis and chlorophyll levels [42,43,44,45,46,47].

A manganese shortage has severe consequences for non-structural carbohydrates, particularly root carbohydrates [48]. Crop quality and quantity are reduced owing to manganese insufficiency, which is caused by poor pollen productivity and a lack of carbohydrates during grain loading. Manganese shortage is similar to magnesium deficiency in that the intercostal areas of the leaves turn yellow.

Manganese insufficiency symptoms develop initially on younger leaves due to the restricted dynamics of these elements in various plant tissues (manganese is not a mobile element). In contrast to manganese, magnesium shortage symptoms are noticed largely in older leaves [8,47,51,52]. Manganese deficiency symptoms in dicotyledonous plants are generally little yellow spots on the leaves; similarly, manganese deficiency symptoms in monocotyledonous plants are taupe and gray-green patches on the base of the leaves. The main sign of insufficiency is a decrease in photosynthetic efficiency, which leads to a general decrease in dry matter production and yield. Manganese deficiency occurs and intensifies according to seasonal circumstances, being more severe in the cold and rainy seasons owing to lower root metabolic activity in manganese absorption. Manganese concentrations in plant tissues have been shown to range between 50 and 150 mg kg^−1^. Manganese essential levels in plant tissues vary according to cultivar, species, and environmental circumstances and have been reported to range from 10 to 50 mg kg^−1^ of dry matter [8,53,54].

### 3.4. Manganese Toxicity

**Figure 3:**
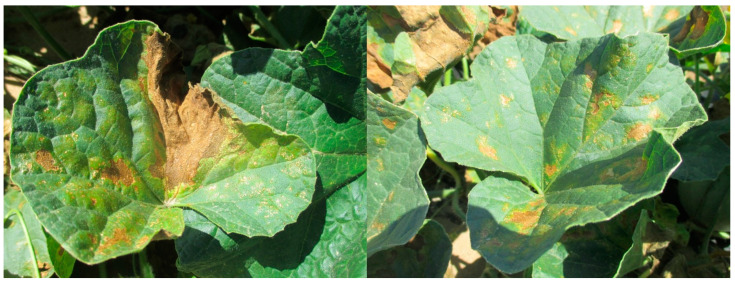
Brown spots and yellowing of leaves due to Mn toxicity in cantaloupe (*Cucumis melo*) [63].

Manganese toxicity in plants (Figure 3) varies according to plant species and environmental factors. Manganese toxicity is a primary factor restricting development in acidic soils. In these soils, excessive amounts of manganese in the leaves inhibit photosynthesis, limiting growth [10,55]. Manganese poisoning causes brown patches on adult leaves and chlorotic dots at the ends of immature leaves. These symptoms show less at higher light intensities than at lower light intensities. Manganese toxicity begins as chlorosis in old leaves and progresses to young leaves [10,55,56,57,58,59,60]. Manganese poisoning symptoms begin at the leaf’s border and progress to regions between leaves, and leaf necrosis spreads with higher toxicity. Manganese poisoning has a greater impact on cell size than on cell number. It causes an uneven distribution of chlorophyll and the build-up of granular starch in chloroplasts. Manganese toxicity may be reduced by using a higher magnesium dose [57,58,61,62].

### 3.5. Manganese’s Impact on Agricultural Production

Manganese shortages in agricultural productivity are most common in alkaline to acidic soils, limiting crop output and yield. Manganese use in the soil, foliar spray, or seed treatment is critical for increased crop production and quality [64]. It promotes carbohydrate synthesis and is necessary for optimal macronutrient usage in plants. Manganese increases the activity of many enzymes that aid in photosynthetic light responses, respiration, and protein synthesis, resulting in greater NPK usage to convert into functional seed carbohydrates [41]. Its foliar application boosts crop production by enhancing photosynthetic efficiency and carbohydrate synthesis, such as starch [64].

Furthermore, manganese plays a crucial metabolic function in nitrate-reducing enzyme activity and the activation of enzymes involved in carbohydrate metabolism; hence, deficits reduce photosynthetic efficiency, resulting in lower crop production and quality [9,19]. In their research, Mousavi et al. [65] found that using manganese and zinc boosted potato yield and improved dry matter storage. In independent studies, Hiller [66] and Brown and Walworth [67] found that foliar sprays of micronutrients such as manganese boosted potato yield and quality. Bansal and Nayyar [68] evaluated the impact of manganese foliar treatments on 10 soybean cultivars and found a considerable improvement in economic and biological yield. Mahler et al. [69] investigated the effects of manganese sulfate on irrigated wheat production and quality and determined that its application boosted wheat yield considerably.

### 3.6. Interactions of Manganese with Other Elements

Manganese absorption varies greatly among plant species, and it is often lower than that of other bivalent cations such as Ca^2+^ and Mg^2+^. Manganese uptake is reduced by magnesium application and liming, with the main reasons being the negative effects of increasing Ca^2+^ and pH. Manganese shares chemical properties with soil alkaline cations such as Ca^2+^ and Mg^2+^, as well as heavy metals such as Zn^2+^ and Fe^2+/3+^; thus, these ions influence manganese uptake and transport in plants [8,70,71,72].

High soil manganese levels have an impact on a plant’s ability to absorb iron, and the same issue (Mn-imposed Fe deficit) may exacerbate the problems caused by manganese toxicity in plants. Furthermore, if the quantity of iron in the soil is too high, plant manganese absorption is inhibited [19,53].

### 3.7. Manganese Availability as a Function of Organic Manure and Manganese Fertilizer Applications

Soil organic matter content, pH value, CaCO_3_ content, and redox conditions all influence manganese (Mn) availability in soils [73]. Submergence causes physical, biological, and chemical changes in soils, all of which are beneficial to rice growth and nutrition. Flooding reduces higher-valent forms of Mn, such as MnO_2_, Mn_2_O_3_, and Mn_3_O_4_, to Mn^2+^, which is available to plants [74]. Green manure is another method for increasing Mn availability in soils. Organic matter releases a variety of organic acids during decomposition, lowering soil pH and increasing the degree of reduction in soils. When green manure is paired with submergence, the reduction of Mn oxides is greater [75].

A similar pattern of distinct types of Mn following a wheat harvest was found due to the lingering influence of green manure as well as farmyard manure (FYM). As a result, an increase in the concentrations of DTPA-extractable, WS+EX, and inorganic Mn suggested that both, green manure and FYM treatment increased Mn availability. The application of manganese sulfate to soil had no discernible impact on distinct Mn factions [76].

## 4. Manganese in Human Nutrition

### 4.1. Manganese in the Human Diet

Mn is an essential nutrient required for many metabolic functions, including normal human development, activation of certain metalloenzymes, energy metabolism, immunological and nervous system function, reproductive hormone function, and antioxidant enzymes that protect cells from free radical damage [77,78]. Mn is also important in regulating cellular energy, bone and connective tissue development, and blood coagulation. Mn serves as a cofactor for several enzymes, including those involved in neurotransmitter production and metabolism [79,80].

Indeed, modest levels of Mn are necessary in human brain development, cellular homeostasis, and the action of various enzymes [81,82,83]. Mn is also thought to be involved in the development of stellate processes in astrocytes as well as the conversion of glutamate in the brain to glutamine, a process carried out by glutamine synthetase [81,82]. Considering the variety of enzymatic processes that require Mn, an inadequate daily supply of the metal is associated with a range of health consequences, including generalized growth impairment, birth defects, reduced fertility, and impaired bone formation, as well as altered lipid, protein, and carbohydrate metabolisms [84,85].

Furthermore, dermatitis, slowed hair and nail growth, decreased serum cholesterol levels, decreased levels of clotting proteins, increased serum calcium and phosphorus concentrations, and increased alkaline phosphatase activity have been reported in humans [77,86,87]. Moreover, various human disorders, including epilepsy, Mseleni joint disease, Down’s syndrome, osteoporosis, and Perthes disease, have been linked to low blood Mn concentrations [88]; nevertheless, the significance of Mn deficiency in these diseases remains unknown. In general, clinical signs require quite severe deficits in dietary Mn supply [89,90]. Furthermore, Mn essentiality in humans is known to vary depending on life stage and sex [91].

### 4.2. Manganese in Food

#### Sources of Manganese

Manganese is found naturally in a variety of foods, including green vegetables, nuts, cereals, and animal products [92]. The most common source of manganese exposure in the general population is food [23,93]. The following are typical manganese content ranges in popular foods (Table 1).

Heavy tea users may consume more manganese than the normal population. Manganese levels in tea may range from 0.4 to 1.3 mg per cup [23]. In 1986, roughly 12% of the adult population in the United States received manganese supplements in addition to food sources [94,95]. The median manganese consumption in these dietary supplements was estimated to be 2.4 mg day^−1^, which is comparable to the amount of the element ingested in the diet (based on data from the Third National Health and Nutrition Estimation Survey, which was conducted in 2001). The risk of manganese overexposure must be balanced against the need for some manganese in the diet, since manganese is an essential mineral that functions as a component of various enzymes and as a participant in a variety of critical physiological processes. Based on a review of human trials, Freeland-Graves et al. [11] proposed a dose range of 3.5–7 mg day^−1^ for adults. Greger [96] offered a range for typical manganese intakes from adult Western and vegetarian diets of 0.7–10.9 mg day^−1^ after evaluating dietary surveys.

The Institute of Medicine’s Food and Nutrition Board established sufficient manganese consumption levels of 2.3 mg day^−1^ for males and 1.8 mg day^−1^ for women. Manganese intake levels of 0.003 mg day^−1^ for infants from birth to 6 months, 0.6 mg day^−1^ for infants from 7 months to 1 year, 1.2 mg day^−1^ for children aged 1–3 years, 1.5–1.9 mg day^−1^ for children aged 4–13 years, and 1.6–2.3 mg day^−1^ for adolescents and adults were also established [92].

### 4.3. Human Manganese Uptake and Distribution

Manganese is absorbed by food, inhalation, and cutaneous permeation, as well as intravenous injection [97]. It is swiftly absorbed in the gastro-intestinal (GI) tract and the lungs before being transported to various organs through blood circulation. The organs with the highest Mn levels in the human body include the liver, pancreas, bones, kidneys, and brain. Although Mn levels in the brain are not the highest among these organs, the brain is the primary target of Mn-induced toxicity since the majority of individuals with Mn poisoning exhibit signs of neurological impairment. Thus, the mechanism through which Mn passes the blood–brain barrier and accumulates in the brain is of particular interest [97].

### 4.4. Manganese Exposure and Absorption Pathways

**Figure 4:**
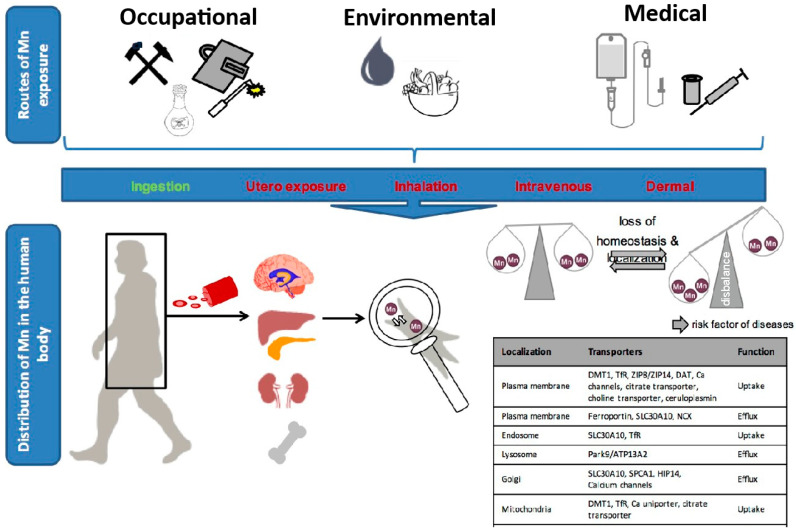
The pathways of human manganese exposure [97].

Figure 4 summarizes the routes of human exposure to manganese, which come from both natural and manmade sources, such as the environment, the workplace, and medical care. Human manganese metabolism and control come from both natural and manmade sources, such as the environment, the workplace, and medical care. There is a proven relationship between Mn pollution of soil and Mn poisoning of the human body. For instance, approximately 80% of Mn emissions originate in the industrial areas of the USA. Additionally, Mn is used in the production of batteries, fertilizers, and animal feed. Over 6.1 thousand metric tons of Mn and almost 73.7 thousand metric tons of Mn-containing compounds were released into the environment from the abovementioned activities (total values up to 2009). However, certain facilities are not required to report Mn release. Thus, this number is probably greater. Approximately 87% of Mn released was deposited into the soil. This supports the fact that the Mn pollutants in soil and water primarily originate from industrial activities and waste materials [23].

Mn enters the bloodstream after absorption and is dispersed via blood circulation. This process is controlled by organs such as the blood, liver, pancreas, kidney, bone, and brain. Mn homeostasis is maintained at the cellular level by both membrane transporters and subcellular transporters. Importers and exporters are two types of membrane transporters. Mn is also distributed and controlled in the endosome, lysosome, Golgi, mitochondria, and nucleus. The loss of Mn homeostasis has been linked to catastrophic brain damage. This “manganism” condition has a comparable neuropathology to Parkinson’s disease (PD) [97].

### 4.5. Ingestion

Mn is most often absorbed via the mouth. The main Mn dietary sources include drinking water, Mn-rich foods, vitamins, supplements, and infant formula. In adults, approximately 3–5% of ingested Mn is absorbed through the GI tract, with females having a higher absorption rate (2.3 mg day^−1^) than males (1.8 mg day^−1^), which may be affected by iron status [92,98,99]. Literature sources mentioning Mn intake differ slightly. As mentioned in the introduction, according to Moss [11], the recommended dose for adults is 3.5 to 7.0 mg per day, whereas O’Neal, S. L., and Zheng, W. [100] mention a range from 2.3 to 8.8 mg per day for Western/USA diets. Currently, there is no official recommended dietary requirement for Mn; however, the estimated safe and acceptable daily food intake of Mn for humans is 2–5 mg day^−1^, and the lowest Mn level in water with a noticeable deleterious impact is 4.2 mg day^−1^ for a 70 kg person [101]. The upper intake level for Mn, which represents the maximum amount unlikely to cause harmful effects, is 11 mg per day for adults [102].

The World Health Organization (WHO) recommends a Mn content of <0.4 mg L^−1^ in drinking water [103]. The human health threshold in the United States is 0.3 mg L^−1^ [104]. In contrast, in Bangladesh, the amount of Mn in the water supply may reach 0.002 m L^−1^, which has been linked to changed classroom behavior in school-aged (8- to 11-year-old) children [105]. Another Mn dietary source that may result in Mn buildup in children is baby formula, particularly soy-based formula [106,107]. However, it may be as much as ten times higher than the permissible amount owing to a lack of maximum Mn levels in formula manufacture [100]. Mn is readily absorbed in the gut and enters cells by passive diffusion or active transport. Mn is transported in human intestinal cells in a biphasic pattern by a saturable mechanism comparable to iron and calcium. It takes around one hour to activate the cellular components (mostly transporters), followed by a gradual increase in Mn absorption after reaching a steady-state condition [108]. Mn absorption in rat intestinal cells is regulated by a high-affinity, low-capacity active transport mechanism [109].

Although it transports other divalent cations, the divalent metal transporter 1 (DMT1) is thought to be primarily responsible for active Mn inflow. Several variables influence Mn absorption. Mn importers are not always Mn-specific transporters since they also control the entrance of other metals, including iron (Fe), copper (Cu), zinc (Zn), calcium (Ca), and so on. As a result, other metals present in biological media (blood, extracellular fluid, etc.) will compete with Mn absorption. Individuals with Fe deficiency are more likely to be poisoned by Mn because Mn absorption in the GI tract increases under low Fe circumstances [110]. Similarly, in iron-deficient rats [111] and pigs [77], the expression of Fe/Mn transporters is changed, and Mn levels in the brain are increased.

Approximately 75% of the Mn in human milk is bound to lactoferrin [78], and excess ferric lactoferrin in brush-border membrane vesicles in the newborn monkey’s small intestine might block absorption of this complexed Mn [79,112]. Furthermore, adding Ca to human milk significantly reduces Mn absorption in both male and female adults. It can be attributed to the calcium’s strong competitive effect, which arises from its role in mineral transport. The effects of other compounds are more variable and may depend on specific conditions or combinations in the diet [113,114,115]. Because of that, adding phytate, phosphate, and ascorbic acid to infant formula, as well as iron and magnesium to wheat bread, probably has no significant effect on Mn absorption [81]. In rats, Mn tends to discharge from the intestine when complexed with albumin or albumin-like proteins, but transferrin-complexed or carrier-free Mn does not. Another aspect that influences Mn absorption is age. Infants and youngsters absorb more Mn from their diets than adults because their bodies need more Mn throughout their development. Mn absorption from milk diets decreased dramatically with age in neonatal rats given human milk, bovine milk, or infant formula [83]. Furthermore, Mn retention was substantially greater in rat pups below 15 days of age (80%) than in older pups (40%) or adults [84]. Despite the fact that the majority of Mn absorption occurs by ingestion, it is regarded as reasonably safe owing to rapid liver clearance.

### 4.6. Prenatal Exposure

In utero, Mn exposure is often overlooked since the actual relationship between Mn exposure and health consequences is unclear. However, there has been an increase in the number of studies examining the relationship between in utero Mn exposure and newborn health. The average Mn concentration (78.8 mg L^−1^) in umbilical cord blood is higher than in the mother’s whole blood (55.0 µg L^−1^), and an inverted U-shaped curve has been observed between Mn levels in mother’s whole blood and birth weights, as well as between Mn levels in umbilical cord blood and birth weights [85]. Other research has shown that both low and high maternal blood Mn levels are related to poor newborn health [86,88].

### 4.7. Inhalation

Most clinically recorded instances of Mn poisoning are the result of occupational exposure. The main route of exposure in occupational Mn poisoning is inhalation of airborne Mn. Industrial workers, particularly miners [89], smelters [90], and welders [116], inhale a considerable quantity of Mn-containing fume and dust, making them the adult group most at risk of Mn-induced toxicity. Mn is absorbed in the lungs and enters the circulation after being inhaled. It may be swiftly transferred to the olfactory bulb and enter the brain through two zinc transporters, ZIP8 and ZIP14, which skip the liver and blood–brain barrier. Mn levels in the lung were higher in rats exposed to 0.0.92 mg MnSO_4_ m^−3^; at 0.9.2 mg MnSO_4_ m^−3^, Mn concentrations in the lung, striatum, and bile were considerably increased [91].

### 4.8. Intravenous and Cutaneous Exposure

Intravenous delivery of drugs containing high quantities of Mn is another Mn exposure route that bypasses GI tract control, resulting in 100% metal absorption [98,117]. Premature newborns, for example, do not absorb adequate nutrition owing to an underdeveloped GI tract or certain disorders. As a result, patients are often supplemented with total parenteral nutrition (TPN) by intravenous injection, which includes several trace elements necessary for life support. Infants on TPN are more vulnerable to Mn toxicity. Furthermore, manganism has been described because of intravenous consumption of methcathinone, which contains manganese dioxide as a by-product from production [118,119]. The absorbed quantity may range between 60 and 180 mg per day, greatly above the typical dietary intake [119,120]. Mn exposure via the skin is also a concern for those who come into contact with organic forms of Mn, such as the gasoline additive methylcyclopentadienyl manganese tricarbonyl (MMT) [23].

## 5. Pharmacokinetics of Manganese

The intricate management of Mn absorption and tissue-specific accumulation is critical for the appropriate regulation of Mn-dependent enzyme activity. Understanding Mn’s importance and toxicity in the brain necessitates knowledge of its control in the periphery. Three primary variables are thought to influence plasma Mn levels. First, since food is the primary source of Mn, careful control of Mn’s gastrointestinal absorption is critical. Second, after Mn absorption and a rise in plasma Mn levels, Mn must be transported to target organs, particularly the liver, to avoid Mn-induced toxicity in the periphery. Finally, Mn from plasma must be removed by shuttling to bile [121]. Thus, homeostatic mechanisms strictly limit Mn absorption and regulate Mn excretion to maintain constant tissue levels despite daily Mn food intake changes. However, high Mn concentrations, such as those seen in industrial contexts, may overpower homeostatic mechanisms, resulting in elevated tissue Mn concentrations. As a result, both pulmonary absorption and particle transport through the olfactory bulb [119,121] may result in Mn deposition in the striatum and cerebellum, as well as nasal epithelial inflammation [100].

Because both metals (Mn^3+^ and Fe^3+^) share binding and absorption through the transferrin (Tf) transporter and the divalent metal transporter 1 (DMT1; also known as the DCT, or divalent cation transporter), it is widely assumed that Fe has a substantial impact on Mn homeostasis. Mn ions (Mn^3+^) bind at the same site as ferric ions (Fe^3+^) on the large glycoprotein molecule mucin, which is known to stabilize the ions and prevent precipitation in the gastrointestinal tract lumen [122]. Furthermore, both metals have been shown to attach to the intercellular metal-binding molecule mobilferrin [123].

Metal ion absorption into enterocytes is known to occur through transmembrane transporters. To enhance Fe absorption during Fe deficiency, the number of transporters in enterocytic membranes is raised [124]. This will definitely enhance Mn absorption, especially in the absence of Fe. Indeed, Fe shortage is linked to increased Mn absorption in the gastrointestinal system as well as increased Mn deposition in the brain in mouse models [125,126]. Furthermore, Mn absorption through the gastrointestinal tract is significantly dependent on the amount of Mn consumed and the net accumulated levels in the plasma. Mn is transported in the large intestine by simple diffusion, whereas it is absorbed in the small intestine via active transport [121]. Mn excretion into bile, on the other hand, is driven by concentration gradients that lead to its movement from liver to bile [127].

Approximately 3–5% of dietary Mn is absorbed as Mn^2+^ and Mn^4+^ in the gastrointestinal system [82]. Mn^2+^ is oxidized to Mn^3+^ in the liver and plasma ceruloplasmin before being transported through the circulation [128,129]. Mn forms close compounds with various ligands [98]. As a result, a number of plasma proteins or ligands, including transglutaminase, beta globulin, albumin, and Tf, have been identified as particular Mn carrier proteins [130,131]. As a consequence, its concentrations in free plasma and tissues are exceedingly low [132].

The Ca^2+^ uniporter sequesters intracellular Mn^2+^ in the mitochondria of the brain and liver [133,134]. Thus, mitochondria are the predominant source of intracellular Mn; yet nuclei have been suggested (but not proven] to preferentially collect this metal [108,135,136]. Furthermore, Mn^2+^ has been found to fragment the Golgi apparatus, suggesting a special function for this compartment in regulating Mn homeostasis [137].

The Ca^2+^/Mn^2+^-ATPases of the secretory route (SPCA) [138] are harbors in the Golgi apparatus and have a high affinity for Mn^2+^ transport [139]. In vivo studies show that brain areas with high SPCA expression have increased Mn^2+^ accumulation after continuous systemic MnCl_2_ infusion in mice [140], and a gain-of-function mutation in SPCA, which specifically enhances Golgi Mn^2+^ transport, improves Mn^2+^-exposed cell survival [141]. Thus, a lack of effective Mn^2+^ detoxification through the Golgi may result in increased Mn^2+^ buildup in the mitochondria, resulting in mitochondrial dysfunction [137].

Mn enters the brain from the blood through cerebral capillaries and/or cerebrospinal fluid. Mn appears to enter the central nervous system primarily through the capillary endothelium at normal plasma concentrations, whereas at high plasma concentrations, transport across the choroid plexus appears to be predominate [142,143], consistent with observations on the rapid appearance and persistent elevation of Mn in this organ [144]. Within one hour of being administered, radioactive Mn is concentrated in the choroid plexus. It is found in the dentate gyrus and cornu ammonis 3 of the hippocampus three days after injection [145].

Mn concentrations in the brain vary depending on the brain region. The globus pallidus in humans and the hypothalamus in rats have the highest Mn levels [81,146,147]. In rats, spectroscopy has revealed that mitochondria in the basal ganglia collect the most Mn [147,148]. Differential metal transporter expression patterns and Mn diffusion constants in different brain areas must account for, at least in part, the asymmetry in Mn buildup across brain regions [149]. The preferential buildup of Mn in the basal ganglia is often linked with manganism, a clinical illness characterized by extrapyramidal symptoms similar to idiopathic Parkinson’s disease. To further understand the foundation of variable Mn accumulation across distinct brain areas, more characterization of absorption and elimination rates, as well as Mn uptake and export mechanisms, is required.

### 5.1. Manganism, a Neurodegenerative Condition

Manganism (also known as locura manganica) is caused by the preferential accumulation of Mn in brain areas rich in dopaminergic (DAergic) neurons (caudate nucleus, putamen, globus pallidus, substantia nigra, and subthalamic nuclei) [12,13,14]. Mn may easily oxidize catecholamines, including dopamine (DA), causing changes in homeostasis in these tissues [150]. The biphasic condition seen in manganism patients is most likely explained by changes in striatal DA levels. An early phase of elevated DA production has been linked to psychotic episodes in psychiatric patients [151]. Catecholamine levels fall as Mn poisoning proceeds, most likely owing to the death of nigrostriatal DAergic neurons, and Parkinson-like symptoms follow [1,92,150]. As a result, in the early phases of manganism, symptoms may be reversed by discontinuing Mn exposure, but manganism is permanent in individuals with motoric abnormalities [136].

### 5.2. Manganese Toxicity Symptoms

Psychiatric symptoms such as emotional liability, mania, compulsive or aggressive behavior, irritability, reduced response speed, hallucinations, feeding and sex disturbances, intellectual deficits, humor changes, sex dysfunctions, and mild motor impairment characterize the early stages of manganism. In situations of established manganism, the characteristic extrapyramidal symptoms (motor symptoms), such as a mask-like face, become evident [152].

## 6. Conclusions

Manganese plays a critical role in both environmental systems and biological organisms, with its influence spanning from the soil to human health. As an essential micronutrient, manganese is vital for plant metabolism, contributing to photosynthesis, enzyme activation, and disease resistance. The environmental cycle of manganese, driven by natural processes and microbial activity, determines its availability in soils and water, directly impacting agricultural productivity.

However, the delicate balance of manganese levels is crucial, as both deficiency and toxicity can have severe consequences. In plants, manganese deficiency can lead to impaired growth and reduced crop yields, while toxicity, often prevalent in acidic soils, can inhibit photosynthesis and stunt development. Similarly, in humans, manganese is essential for numerous physiological processes, but overexposure, particularly in occupational settings, can lead to neurodegenerative conditions such as manganism.

Understanding the complex interactions between manganese’s environmental cycling, its role in plant physiology, and its impact on human health underscores the importance of managing manganese levels in agriculture and industry. Future research should continue to explore these connections, aiming to optimize manganese use in agriculture while minimizing the risks of toxicity in both plants and humans. This holistic approach is essential for sustainable agricultural practices and public health safety.

## Figures and Tables

**Table 1:** Average manganese concentrations in different types of food [23].

Type of Food	Range of Mean Concentrations (mg kg^−1^)
Nuts and nut products	18.2–46.8
Grains and grain products	0.42–40.7
Legumes	2.24–6.73
Vegetables and vegetable products	0.42–6.64
Fruits	0.20–10.4
Infant foods	0.17–4.83
Fruit juices and drinks	0.05–11.5
Desserts	0.04–7.98
Meat, poultry, fish and eggs	0.10–3.99
Mixed dishes	0.69–2.98
Condiments, fats and sweeteners	0.04–1.45
Beverages (including tea)	0.00–2.09
Soups	0.19–0.65
Milk and milk products	0.02–0.49
